# Effects of Modified Low-Density Lipoproteins and Fenofibrate on an Outer Blood-Retina Barrier Model: Implications for Diabetic Retinopathy

**DOI:** 10.1089/jop.2020.0068

**Published:** 2020-12-11

**Authors:** Dongxu Fu, Jeremy Y. Yu, Anna R. Connell, Michelle B. Hookham, Rebecca H. McLeese, Timothy J. Lyons

**Affiliations:** ^1^ Wellcome-Wolfson Institute for Experimental Medicine, School of Medicine, Dentistry and Biomedical Sciences, Queen's University Belfast, Northern Ireland, United Kingdom.; ^2^ Division of Endocrinology, Diabetes and Metabolic Diseases, Medical University of South Carolina, Charleston, South Carolina, USA.; ^3^ Diabetes Free SC, BlueCross BlueShield of South Carolina, Columbia, South Carolina, USA.

**Keywords:** blood-retina barrier, diabetic retinopathy, fenofibrate, lipoprotein, oxidized LDL, retinal pigment epithelium

## Abstract

***Purpose:*** There is a lack of treatment for early diabetic retinopathy (DR), including blood-retina barrier (BRB) breakdown. The robust clinical benefit of fenofibrate in DR provides an opportunity to explore disease mechanisms and therapeutic targets. We have previously found that modified lipoproteins contribute to DR and that fenofibrate protects the inner BRB. We now investigate (1) whether modified lipoproteins elicit outer BRB injury and (2) whether fenofibrate may alleviate such damage.

***Methods:*** Human retinal pigment epithelium ARPE-19 cells were cultured in semipermeable transwells to establish a monolayer barrier and then exposed to heavily oxidized, glycated low-density lipoprotein (HOG-LDL, 25–300 mg/L, up to 24 h) versus native (N)-LDL. Transepithelial electric resistance (TEER) and FITC-dextran permeability were measured. The effects of fenofibrate, its active metabolite fenofibric acid, and other peroxisome proliferator-activated receptor (PPARα) agonists (gemfibrozil, bezafibrate, and WY14643) were evaluated, with and without the PPARα antagonist GW6471 or the adenosine monophosphate-activated protein kinase (AMPK) inhibitor Compound C.

***Results:*** HOG-LDL induced concentration- and time-dependent barrier impairment, decreasing TEER and increasing dextran leakage, effects that were amplified by high glucose. Fenofibric acid, but not fenofibrate, gemfibrozil, bezafibrate, or WY14643, attenuated barrier impairment. This effect was reversed significantly by Compound C, but not by GW6471.

***Conclusions:*** Modified lipoproteins elicited outer BRB injury in an experimental model, which was reduced by fenofibric acid through a PPARα-independent, AMPK-mediated mechanism. These findings suggest a protective role of fenofibric acid on the outer BRB in diabetic retina.

## Introduction

Fenofibrate has been used clinically for decades to manage hypertriglyceridemia, and its action is mediated by the peroxisome proliferator-activated receptor alpha (PPARα), a nuclear receptor that regulates the genes for fatty acid catabolism. At least two large randomized controlled clinical trials, the *Fenofibrate Intervention and Event Lowering in Diabetes (FIELD)* and the *Action to Control Cardiovascular Risk in Diabetes (ACCORD)*, have independently revealed robust therapeutic effects of fenofibrate on diabetic retinopathy (DR): in both, there was ∼30% reduction in DR progression in patients taking the medication.^1,2^ Intriguingly, in both studies, the retinal benefits were independent of plasma lipid lowering effects, suggesting that the drug may act within the retina, and possibly by a mechanism independent of PPARα.^3,4^ Thus, understanding its retinal mechanisms of action may reveal new opportunities to prevent or treat DR, and may potentially identify new pathogenic mechanisms.

We have previously explored the effects of fenofibrate, given by both oral and intravitreal routes, in rodent models of type 1 diabetes and oxygen-induced retinopathy: it reduced retinal vascular leakage, decreased inflammatory mediators, and inhibited neovascularization in a PPARα-dependent manner.^5^ Its efficacy following intravitreal administration suggested a local action within the retina.

The outer blood-retina barrier (BRB) is formed by the retinal pigment epithelium (RPE); its compromise is detrimental to neural retina.^6^ Although most research has focused on the readily visualized inner BRB, evidence indicates that the outer BRB is also deficient in DR.^7^ Previously, we have reported the accumulation of modified low-density lipoprotein (LDL) in diabetic retinas, which is detectable before clinical DR onset, and increases thereafter to an extent commensurate with disease severity.^8^ We have also shown that modified LDL is injurious to several retinal cell types, including RPEs.^9–14^ We therefore hypothesized that extravasated, modified LDL might play an important role in DR development,^15^ and might act, in part, by injuring not only the inner BRB but also the outer BRB. We also hypothesized that the protective effects of fenofibrate in DR include preservation of outer BRB integrity and reduction of vascular endothelial growth factor (VEGF) and inflammatory mediator expression.

To test these hypotheses, we established a model of outer BRB injury using a cultured monolayer of human RPE cells exposed to *ex vivo*-modified human LDL. We tested fenofibric acid (the active moiety of fenofibrate) as a protective agent and determined whether or not its action was mediated by PPARα. We included other fibrate drugs for comparison.

## Methods

### Human lipoprotein preparation

Preparation of human native (N-) and “highly oxidized, glycated” (HOG-) LDL was described previously.^16,17^ Briefly, N-LDL (density 1.019–1.063) was isolated by sequential ultracentrifugation of pooled plasma from 4 to 6 healthy fasted volunteers. Glycated LDL was prepared by incubating LDL with freshly prepared 50 mM glucose (72 h at 37°C) under antioxidant conditions (1 mM diethylenetriaminepentaacetic acid [DTPA] with 270 μM EDTA, under nitrogen). HOG-LDL was prepared by oxidizing glycated LDL in the presence of 10 μM copper chloride (24 h, 37°C), followed by repeated dialysis (24 h at 4°C). LDL protein concentration was determined by the BCA assay (Pierce, Rockford, IL). LDL preparations were further characterized by fluorescence (Ex 360 nm; Em 430 nm), agarose gel electrophoresis (Paragon Lipogel; Beckman, Fullerton, CA), and absorbance at 234 nm. They were stored in the dark under nitrogen gas at 4°C, and used within 6 weeks. Experiments were repeated using different batches of lipoprotein preparations.

### Cell culture

ARPE-19 cells (American Type Culture Collection, Manassas, VA) at passages 3–15 were maintained in DMEM supplemented with 10% fetal bovine serum (Sigma-Aldrich, St. Louis, MO) at 37°C under 5% CO_2_ and 95% air. A confluent monolayer barrier was established following the previously published methods.^18,19^ Cells were seeded onto 24-well semipermeable transwell plates and cultured in growth medium for 18–21 days, when a tight barrier was formed; the medium was refreshed every 2–3 days. The cells were then switched to serum-free medium (SFM) overnight before treatments with lipoproteins and/or other agents. Conventional cell culture was also used to complement the barrier model for mechanistic studies. Fenofibrate, fenofibric acid, gemfibrozil, bezafibrate, WY14643, GW6471, and Compound C (all from Sigma-Aldrich, St. Louis, MO) were spiked into culture media or into transwell inserts (i.e., apical side of the RPE barrier). Four-hydroxynonenal (4-HNE; Cayman Chemical, Ann Arbor, MI) was assessed as a potentially convenient means to simulate the effects of HOG-LDL. The blocking antibody for lectin-type oxidized LDL receptor 1 (LOX-1) was obtained from Thermo-Fisher Scientific (Raleigh, NC).

### Transepithelial electric resistance

Transepithelial electric resistance (TEER) was measured using a Millicell epithelial voltmeter (Millipore, Billerica, MA) with STX100C electrodes (World Precision Instruments, Sarasota, FL), as per manufacturers' instructions. Net TEER was calculated by subtracting background resistance of the transwell insert alone from the value obtained with the insert containing RPEs.

### Barrier permeability assay

Fluorescein isothiocyanate (FITC)-dextran (10 kDa; Sigma-Aldrich, Saint Louis, MO) was added to the apical compartment of RPE cell monolayer at 100 mg/mL. Following 1 h of incubation at 37°C, 200 μL medium from the basolateral compartment was transferred onto a flat-bottom 96-well plate. Barrier permeability was determined by fluorescence spectroscopy at Ex 485 nm and Em 528 nm (VICTOR3 microplate reader; PerkinElmer, Waltham, MA). All experiments were performed in triplicate.

### Western blotting

Cells grown on standard 6-well plates were homogenized with the complete lysis buffer (Roche Diagnostics, Indianapolis, IN), with protein concentrations measured by the BCA assay (Thermo-Fisher Scientific, Rockford, IL). Thirty micrograms protein was loaded onto each lane of a 12% sodium dodecyl sulfate–polyacrylamide gel. Proteins were separated by electrophoresis, transferred to nitrocellulose membranes, and probed with primary antibodies at a dilution of 1:1000 unless otherwise indicated. Antibodies against β-actin (1:3000), VEGF, p-AMPK, and total AMPK were from Cell Signalling Technology (Danvers, MA); antibodies against LOX-1 were from Abcam (Cambridge, MA). β-Actin was used as a loading control. The membranes were developed by Pierce ECL Western Blotting Substrate (Thermo-Fisher Scientific, Raleigh, NC), with the image captured with a UVP BioSpectrum Imaging System (UVP, Upland, CA). Intensities of individual bands were quantified by densitometry (ChemStudio Plus, UVP, Upland, CA), with the background subtracted from calculated areas. All experiments were repeated three times independently.

### VEGF measurement

Supernatant VEGF levels were assayed in duplicate by a DuoSet ELISA kit (R&D Systems, Minneapolis, MN) as per manufacturer's manual.

### Quantitative real-time PCR

RNA was extracted from the cells (RNeasy Mini Kit; Qiagen, Valencia, CA) and cDNA was synthesized using Superscript III (Invitrogen, Paisley, United Kingdom). Semiquantitative real-time PCR was performed for VEGF, intercellular adhesion molecule-1 (ICAM-1), and the reference gene β-actin. Relative gene expression was calculated using the ΔΔCt method.

### Data analysis

Data were expressed as mean ± SD. Statistical significance was determined by Student *t*-test or one-way ANOVA, followed by *post-hoc* Bonferroni's test as appropriate (Prism 5; Graphpad, La Jolla, CA). *P* values ≤0.05 were considered significant.

## Results

### Effects of modified human LDL on RPE barrier

**FIG. 1.**
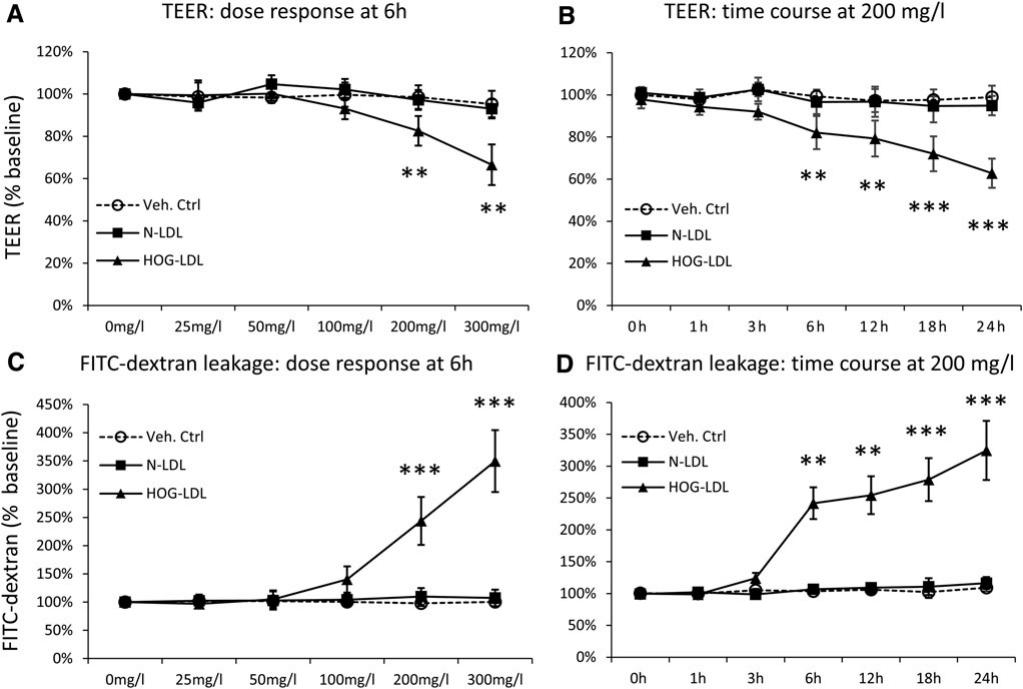
HOG-LDL decreased TEER and induced FITC-dextran leakage in an RPE barrier model. ARPE-19 cells were cultured in transwell plates for 18–21 days to form a confluent monolayer and then treated with SFM for 18 h to achieve quiescence. Cells were then exposed to HOG-LDL or N-LDL at concentrations and time points indicated, versus PBS as a vehicle control. TEER and FITC-dextran leakage were measured; no-treatment and 0-h values served as the baseline, respectively. TEER responses are shown in exposure to **(A)** HOG- versus N-LDL (0–300 mg/L) at 6 h and **(B)** HOG- versus N-LDL (200 mg/L) over 0–24 h. FITC-dextran leakage responses are shown in exposure to **(C)** HOG- versus N-LDL (0–300 mg/L) at 6 h, and **(D)** HOG- versus N-LDL (200 mg/L) for 0–24 h. Data are presented as percentages relative to the baseline (mean ± SD, *n* = 3). **P < 0.01 and ***P < 0.001 versus vehicle control. TEER, transepithelial electric resistance; HOG-LDL, heavily oxidized, glycated low-density lipoprotein; SFM, serum-free medium; FITC, fluorescein isothiocyanate.

We first established a confluent monolayer barrier model of ARPE-19 cells on porous transwell plates, which was confirmed by visual inspection of live culture under phase-contrast microscopy and by ZO-1 tight junction protein staining. The baseline TEER measures were 112.3 ± 20.4 Ω.cm^2^ (*n* = 27), consistent with the literature data for this model^20^; this TEER range was also in line with the reported values (36–148 Ω.cm^2^) from the isolated adult human RPE-choroid tissue.^21^ Treatment of HOG-LDL (25–300 mg/mL protein, 6 h), versus N-LDL or phosphate-buffered saline (PBS), elicited concentration-dependent TEER reductions (Fig. 1A). When tested at a fixed concentration of 200 mg/mL, HOG-LDL also induced time-dependent barrier dysfunction, while N-LDL had no effect (Fig. 1B). This was confirmed by the FITC-dextran permeability assay: HOG-LDL induced significant leakage in both a concentration- and time-dependent manner relative to N-LDL or vehicle control (Fig. 1C, D).

### High glucose potentiated HOG-LDL-induced RPE barrier breakdown

**FIG. 2.**
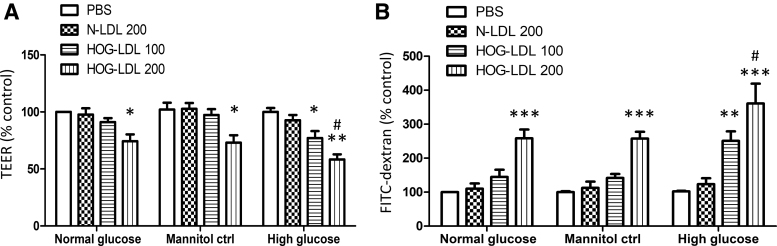
High glucose potentiated HOG-LDL-induced RPE barrier breakdown. Quiescent monolayer ARPE-19 cells were pretreated with normal glucose (5.5 mM), high glucose (25 mM), or mannitol (at a comparable concentration as an osmotic control) for 24 h, and then treated with N-LDL (200 mg/L) or HOG-LDL (100 or 200 mg/L) for 6 h. PBS in normal glucose served as the baseline control. **(A)** TEER and **(B)** FITC-dextran leakage were measured. Data are presented as percentages relative to the baseline control (mean ± SD, *n* = 3). *P < 0.05, **P < 0.01, and ***P < 0.001 versus N-LDL. ^#^*P* < 0.05 versus the normal glucose or mannitol control.

To investigate whether hyperglycemia may modulate the effects of modified lipoproteins, we assessed barrier function in the presence of 5.5 and 25 mM glucose to simulate normoglycemia and hyperglycemia, respectively. Cells were pretreated with glucose (or with mannitol to reproduce the osmotic stress of 25 mM glucose) for 24 h, followed by lipoprotein challenge for 6 h. As above, in the presence of basal level of 5.5 mM glucose, TEER was decreased by HOG-LDL in a concentration-related manner, reaching statistical significance at 200 mg/L. In the presence of 25 mM glucose (but not when mannitol was used to simulate the same increase in osmotic stress), HOG-LDL elicited a greater reduction of TEER, statistically significant at both 100 and 200 mg/L (Fig. 2A). This suggests that, while high glucose had no effect by itself, it sensitized RPEs to HOG-LDL-induced injury, and that this effect was not mediated by increased osmotic stress. Again, the FITC-dextran permeability assay showed similar results: if glucose concentrations were elevated, injurious effects of HOG-LDL on barrier function were amplified (Fig. 2B).

### Four-HNE mimicked HOG-LDL effects on RPE barrier

**FIG. 3.**
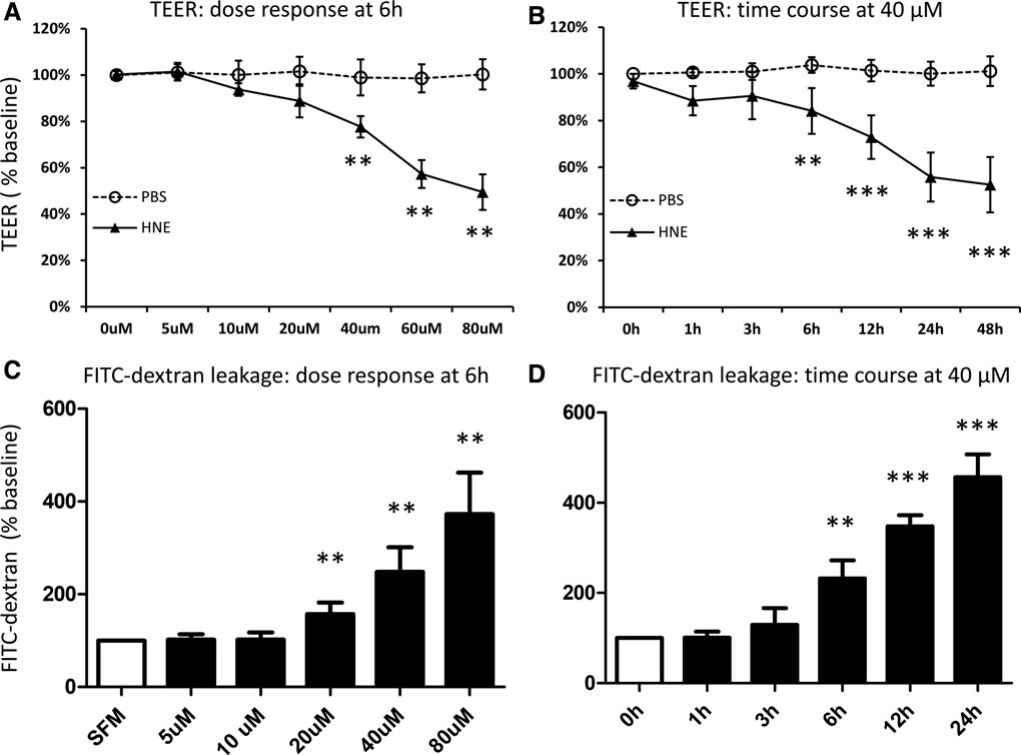
4-HNE mimicked HOG-LDL effects on the RPE barrier model. Quiescent monolayer ARPE-19 cells were exposed to 4-HNE (0–80 μM) with PBS as a vehicle control. Concentration- and time-course responses for TEER and FITC-dextran leakage were measured; no-treatment and 0-h group values served as the baseline, respectively. TEER responses are shown in exposure to **(A)** 4-HNE (0–80 μM, 6 h) and **(B)** 4-HNE (40 μM, 0–48 h). FITC-dextran leakage responses are shown in exposure to **(C)** 4-HNE (0–80 μM, 6 h) and **(D)** 4-HNE (40 μM, 0–24 h). Data are presented as percentages relative to SFM (mean ± SD, *n* = 3). **P < 0.01 and ***P < 0.001 versus vehicle control. 4-HNE, 4-hydroxynonenal.

Four-HNE is a major reactive carbonyl-containing fatty acid oxidation product in oxidized LDL (ox-LDL) particles, and has been previously shown to replicate some of the effects of HOG-LDL, including those on retinal pericytes, Müller cells, and RPEs.^10–12^ In this study, we treated RPEs with commercially available 4-HNE in place of HOG-LDL, measuring barrier function as above. The effect of 4-HNE (5–80 μM) largely resembled that of HOG-LDL: it decreased TEER (Fig. 3A, B) and increased FITC-dextran leakage (Fig. 3C, D) in a concentration- and time-dependent manner.

### LOX-1 receptors were involved in HOG-LDL-induced RPE barrier dysfunction

**FIG. 4.**
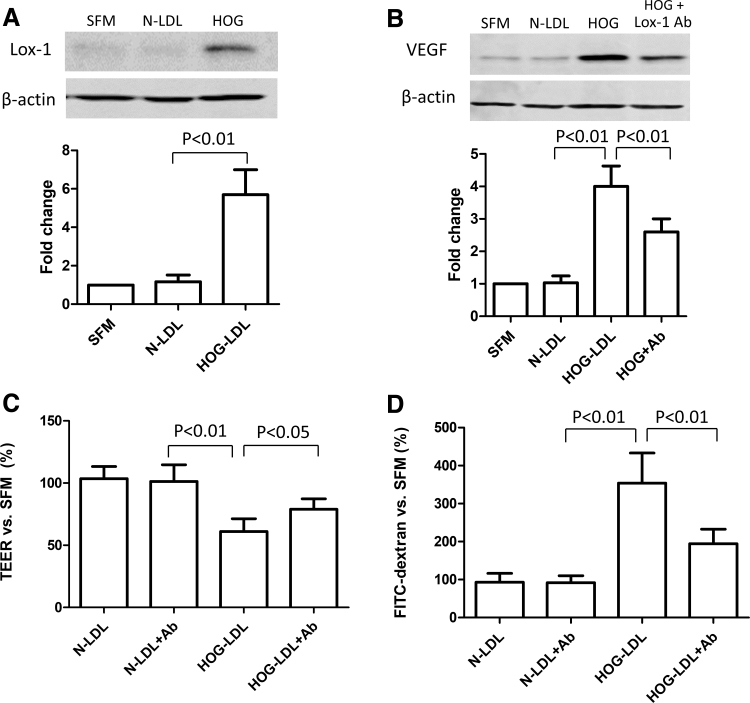
LOX-1 was involved in HOG-LDL-induced RPE barrier dysfunction. **(A, B)** ARPE-19 cells were cultured in 6-well plates for 48 h to reach 80% confluence, and then kept quiescent in SFM for 18 h. Cells were pretreated with versus without a, LOX-1 blocking antibody (50 mg/L, 1 h), and then exposed to N- or HOG-LDL treatment (200 mg/L, 12 h); protein levels of LOX-1 and VEGF were determined by Western blotting and densitometry. **(C, D)** Experiments were also conducted in the monolayer ARPE-19 barrier model in transwells to determine permeability using TEER and FITC-dextran leakage assays. Data are presented as percentages versus SFM (mean ± SD, *n* = 3 or 5).

LOX-1 as a major scavenger receptor has been implicated in ox-LDL-mediated pathogenesis. In this study, HOG-LDL, but not N-LDL (both at 200 mg/L), induced significant LOX-1 protein expression in cultured RPEs (Fig. 4A). To further understand the role of LOX-1, in both standard cell culture and the RPE monolayer model, we pretreated the cells with an LOX-1 blocking antibody before HOG-LDL treatment. LOX-1 blockade reduced VEGF protein expression (Fig. 4B), which has a critical role in RPE barrier maintenance, and attenuated HOG-LDL-induced barrier functional impairment (Fig. 4C, D). These data suggest that LOX-1 is implicated in HOG-LDL-induced RPE barrier damage.

### Fenofibric acid alleviated HOG-LDL- and 4-HNE-induced RPE barrier impairment

**FIG. 5.**
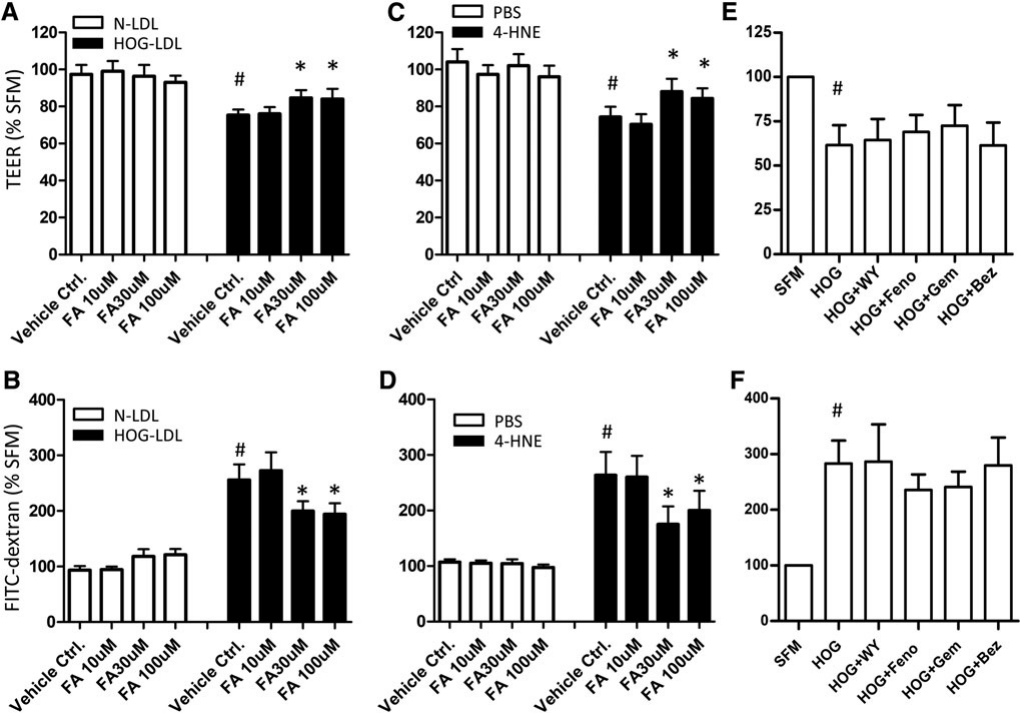
Fenofibric acid attenuated HOG-LDL- and 4-HNE-induced RPE barrier dysfunction. Monolayer ARPE-19 cells were pretreated with/without FA for 1 h at the concentrations indicated, or fenofibrate (Feno, 30 μM), WY14643 (WY, 10 μM), gemfibrozil (Gem, 30 μM), or bezafibrate (Bez, 30 μM), and then challenged with N- or HOG-LDL (200 mg/L, 6 h) or 4-HNE (40 μM, 6 h). **(A, C, E)** TEER; **(B, D, F)** FITC-dextran leakage. Data are presented as percentages relative to vehicle control. Mean ± SD, *n* = 3 or 5. ^#^*P* < 0.05 versus N-LDL or PBS control (*open bars*), indicating barrier impairment elicited by HOG-LDL. *P < 0.05 versus HOG-LDL or 4-HNE without drug treatments (*solid bars*), indicating barrier-protective effect by the drugs. FA, fenofibric acid; RPE, retinal pigment epithelium.

Fenofibrate is an ester prodrug that undergoes hydrolysis by hepatic or plasma esterases to form the active metabolite fenofibric acid. Considering the unknown efficiency of hydrolysis *in vitro*, we tested fenofibric acid in our experiments. We pretreated RPEs with fenofibric acid (10, 30, and 100 μM), before exposing them to HOG-LDL (200 mg/L; vs. N-LDL) or 4-HNE (40 μM; vs. PBS) for 6 h. Fenofibric acid significantly attenuated HOG-LDL-induced TEER reduction and FITC-dextran leakage at 30–100 μM (Fig. 5A, B), suggesting a protective effect. It was also effective against 4-HNE-induced barrier breakdown (Fig. 5C, D). To determine whether this is a class action, we tested fenofibrate (the prodrug) and three additional PPARα agonists (gemfibrozil, bezafibrate, and WY14643): to our surprise, none had significant effects in preserving barrier function (Fig. 5E, F).

### AMPK, but not PPARα, mediated the effect of fenofibric acid

**FIG. 6.**
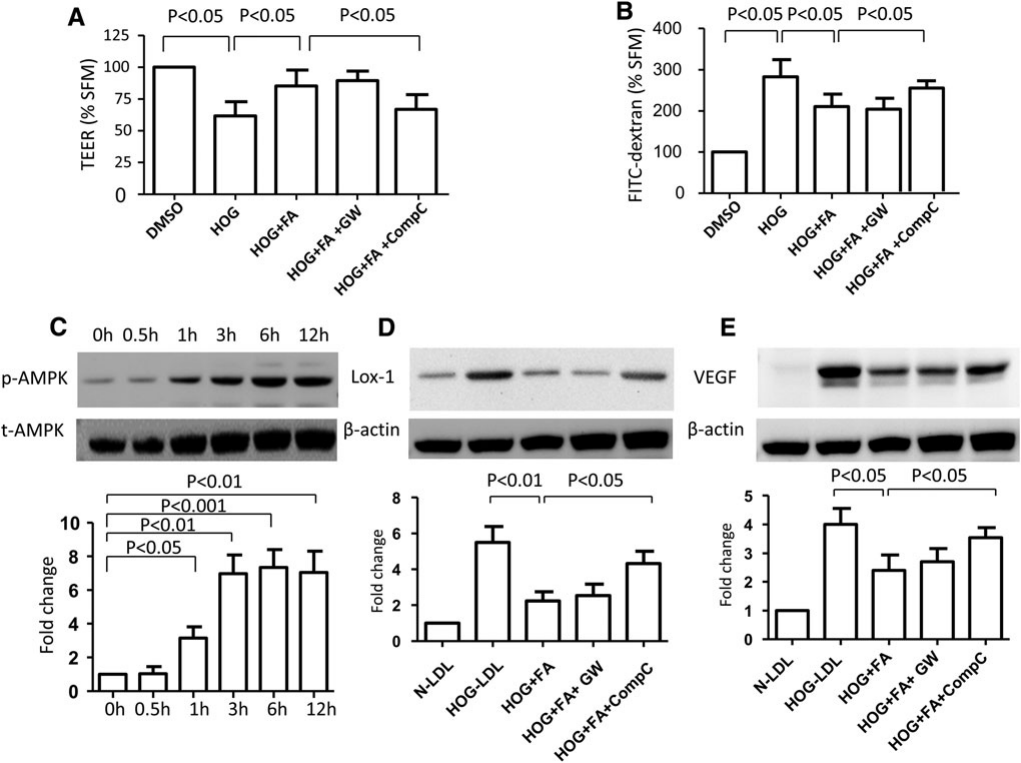
AMPK, but not PPARα, was implicated in the protective role of fenofibric acid on HOG-LDL-induced RPE barrier dysfunction. **(A, B)** Quiescent monolayer ARPE-19 cells were pretreated with vehicle control (DMSO), fenofibric acid (FA, 30 μM), FA (30 μM) + GW6471 (GW, 10 μM), or FA (30 μM) + Compound C (CompC, 10 μM) for 1 h, and then challenged with HOG-LDL (200 mg/L) for 6 h. TEER and FITC-dextran leakage were measured accordingly. Data are presented as percentage versus vehicle control (mean ± SD, *n* = 5). There was no significant difference between the HOG+FA+CompC treatment versus HOG-LDL treatment alone. **(C)** ARPE-19 cells were treated with FA (30 μM) for 0–12 h; p-AMPK and total AMPK were detected by Western blotting and densitometry. **(D, E)** ARPE-19 cells were pretreated with FA (30 μM), FA (30 μM) + GW6471 (10 μM), or FA (30 μM) + Compound C (10 μM) for 1 h, and then exposed to HOG-LDL (200 mg/L) for 6 h; N-LDL (200 mg/L) served as a control. Protein levels of LOX-1 and VEGF were detected by Western blotting and densitometry. Data are presented as percentages versus N-LDL (mean ± SD, *n* = 3 or 5). There was no significant difference between the HOG+FA+CompC treatment versus HOG-LDL treatment alone. VEGF, vascular endothelial growth factor; FA, fenofibric acid.

**FIG. 7.**
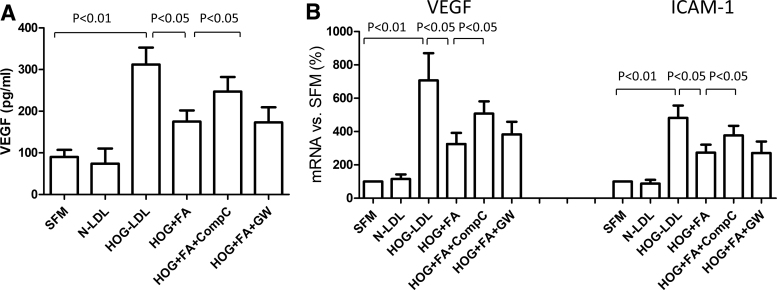
VEGF protein and mRNA expression, and ICAM-1 mRNA expression. ARPE-19 cells were treated with N-LDL or HOG-LDL (200 mg/L, 6 h), with/without pretreatment with FA (30 μM), FA (30 μM) + Compound C (CompC, 10 μM), or FA (30 μM) + GW6471 (GW, 10 μM) for 1 h. **(A)** Supernatant VEGF protein was measured by ELISA; **(B)** mRNA expression for VEGF and ICAM-1 was determined by real-time PCR. Data are presented as percentages versus SFM (mean ± SD, *n* = 3 or 5). There was no significant difference between the HOG+FA+CompC treatment versus HOG-LDL treatment alone.

To explore the mechanism underlying the protective effect of fenofibric acid, we evaluated our barrier model in the presence versus absence of the PPARα antagonist GW6471 and the AMPK inhibitor Compound C: the effect of fenofibric acid was blocked by Compound C, but not by GW6471 (Fig. 6A, B). Fenofibric acid activated AMPK (measured by p-AMPK vs. total AMPK) in a time-dependent manner, beginning as early as 1 h and reaching a plateau after 3 h (Fig. 6C). GW6471 did not reverse the effect of fenofibric acid on LOX-1 (Fig. 6D) or VEGF expression (Fig. 6E), whereas Compound C did so. Overall, these data suggest a PPARα-independent action of fenofibric acid. Consistent with this, we found that fenofibric acid attenuated HOG-LDL-induced VEGF secretion from RPEs in an AMPK-dependent manner (Fig. 7A). Furthermore, HOG-LDL induced mRNA expression of both VEGF and ICAM-1 (Fig. 7B) (note that ICAM-1 protein was undetectable by ELISA in the supernatant): these effects were inhibited by fenofibric acid, and again, Compound C, but not GW6471, attenuated the effects of fenofibric acid.

## Discussion

Since the unexpected discovery of the therapeutic value of fenofibrate for DR in two pivotal clinical trials,^1,2^ there has been great interest in understanding the underlying mechanism(s) of action. One intriguing observation was that the retinal benefits were independent of effects on plasma lipids and lipoproteins. Thus, the drug may affect lipid metabolism within the retina, a compartment normally protected by tight BRBs, in a manner that is different from that in the periphery; alternatively, it may act through a non-PPARα mechanism. Historically, plasma lipoproteins have been linked with DR in epidemiological studies,^22–24^ but the associations are too weak to define individual risk.^25,26^ It is known that the retina has an intricate physiological lipid system, which shares similarities with other organs, but also has unique features.^27^ In addition, we have observed pathological accumulation of oxidatively modified lipoproteins in the retinas of diabetic patients (presumably after extravasation due to leaking BRBs with ensuing modification accelerated by the diabetic milieu). This “invasion” of the retina occurs even in diabetic patients with no clinically evident DR, and in those with retinopathy, to an extent commensurate with the severity of DR.^8^ In plasma, ox-LDL constitutes only a small percentage of the total LDL pool,^28^ but its local tissue concentration can be substantially higher following extravasation,^8,29^ and in these locations, antioxidant capacity is finite. Extravasated and modified lipoproteins, we hypothesize, may represent an important mechanism in DR pathogenesis and a potential target for fenofibrate, especially since there is evidence that the drug can modulate ox-LDL and its scavenger receptors.^30–32^

To test these hypotheses, we investigated the effect of *ex vivo* modified human LDL on an *in vitro* model of human RPE barrier, and the potential protective role of fenofibrate and its active metabolite fenofibric acid. We found that (1) human modified LDL (and one of its key toxic components, 4-HNE) elicited concentration- and time-dependent RPE barrier impairment, which was amplified by high glucose; this effect was partially mediated by the scavenger receptor LOX-1; (2) fenofibric acid mitigated barrier impairment; and (3) this effect appeared to be mediated by the activation of AMPK, but not PPARα.

The injurious effect of modified LDL on the outer BRB is not surprising, in view of its toxicity toward a variety of human retinal cells, including RPEs, which we and others have demonstrated.^9–14,33–35^ Kim et al.^34^ reported that ox-LDL was able to induce RPE senescence and compromise barrier integrity as measured by a fluorescence dye. Our data confirmed their observation; furthermore, we showed concentration- and time-dependent barrier leakage caused by both modified LDL and one of its components, 4-HNE, using two cross-validating methods (TEER and FITC-dextran permeability). Thus, the data support the notion that modified LDL-induced RPE barrier leakage may contribute to the pathogenesis of DR. The congruent effects of modified LDL and 4-HNE also suggest that readily available 4-HNE may serve as a convenient surrogate for modified LDL. The utility of such a model may extend to other retinal diseases, such as age-related macular degeneration^35,36^ and retinitis pigmentosa.^37^

We observed a synergy between high glucose and modified LDL: a high concentration of glucose (25 mM) alone did not cause evident RPE barrier leakage; however, it potentiated the injurious effects of HOG-LDL. This is in agreement with another recent study, in which we showed that intravitreal injection of HOG-LDL elicited significant injury to the diabetic retina, but only mild, transient inflammation in the retina of nondiabetic mice: this suggested that diabetes confers susceptibility to retinal injury imposed by modified LDL.^15^ Mannitol, as an “osmotic control,” did not potentiate the effect of HOG-LDL, suggesting a metabolic effect of high glucose.

RPEs internalize ox-LDL by scavenger receptors, including CD36 (cluster of differentiation 36) and LOX-1.^38^ Although LOX-1 is constitutively expressed at low levels in RPEs,^35,38^ it is upregulated in response to pathological stimuli such as inflammation.^39^ In this study, we found that LOX-1 protein expression was significantly enhanced by HOG-LDL, and an LOX-1 blocking antibody attenuated HOG-LDL-induced barrier impairment and VEGF upregulation. A similar process has been implicated in atherogenesis, in which cells internalize ox-LDL by LOX-1, and LOX-1 blockade is protective.^40^ Although we did not investigate the expression of LOX-1 *per se*, others have reported a reduction of LOX-1 expression following its blockade by either neutralizing antibodies or chemical compounds in various scenarios.^41,42^

Fenofibrate is a prodrug that is cleaved by tissue and plasma esterases to its active form, fenofibric acid; the two have differing pharmacodynamic properties, likely accounting for some of the discrepancies in the literature.^43,44^ We found a protective effect of fenofibric acid on HOG-LDL-induced RPE barrier leakage at a clinically relevant concentration of 30 μM; this effect was not shared by the parent drug, fenofibrate. Several other fibrates, including gemfibrozil, bezafibrate, and WY14643, all non-prodrug PPARα agonists, also failed to show protection. The action of fenofibric acid appeared to be mediated by AMPK activation, being inhibited significantly by the AMPK inhibitor Compound C, but not by the PPARα inhibitor GW6471. Together, these data support a PPARα-independent mechanism of action. Fenofibric acid also suppressed HOG-LDL-induced overexpression of LOX-1, VEGF, and ICAM-1, suggesting that its beneficial effects may be partially mediated by LOX-1. Again, consistent with the functional barrier data, the effects of fenofibric acid on LOX-1, VEGF, and ICAM-1 were reversed by Compound C, but not by GW6471.

Increasing evidence indicates that fenofibrate and its active metabolite fenofibric acid have pleotropic effects through both PPARα-dependent and PPARα-independent mechanisms. In porcine retinal arterioles, fenofibrate elicited endothelium-dependent dilation that was unrelated to PPARα,^45^ and several fibrate drugs have shown effects on inducible nitric oxide synthase and NF-κB expression in human mesangial cells devoid of functional PPARα.^46^ AMPK activation appears to be a common PPARα-independent mechanism in endothelial cells, retinas, and other vascular tissues. Kim et al.^47^ found that in human retinal endothelial cells, fenofibrate prevented apoptosis induced by serum deprivation through an AMPK-dependent, non-PPARα pathway. In that study, fenofibrate activated AMPK and stimulated VEGF mRNA expression: both effects were inhibited by Compound C, but neither the PPARα antagonist MK886 nor the PPARα agonist WY14643 had any effect. In human glomerular endothelial cells, activation of AMPK by fenofibrate diminished inflammation and death induced by advanced glycation products and high glucose, but bezafibrate was not effective.^48^ However, fenofibrate did not activate AMPK or provide protection to hepatocytes, suggesting that this mechanism is likely to be tissue specific.^48^ Intriguingly, Villarroel et al.^49^ reported that fenofibric acid prevented interleukin 1β-induced RPE barrier leakage by inhibiting AMPK. In their more recent study, fenofibric acid was further found to prevent interleukin 1β-induced RPE barrier leakage through PPARα-mediated inhibition of NF-κB activity.^50^ Thus, it is possible that fenofibric acid may achieve its efficacy in RPE barrier protection through both PPARα-dependent and PPARα-independent mechanisms, depending on the actual stimuli or matrices involved. In the case of ox-LDL, generally in line with our present results, the protective role of AMPK has previously been shown in macrophages, where its activation attenuated ox-LDL uptake through the PP2A/NF-κB/LOX-1 pathway.^51^

Translational research for outer BRB using *in vitro* systems remains a challenge. While the widely used ARPE-19 monolayer model has been shown to exhibit polarity and barrier properties under culture conditions and durations similar to this study, recent evidence suggests that this may represent an early differentiated stage and full maturity may only be achieved after 4 months in culture.^19^ ARPE-19 cells have also been thought to resemble a pathologic or aged RPE.^52^ Although the RPE biomarkers such as RPE65 and cellular retinaldehyde binding protein (CRALBP) are expressed in APRE-19 cells, the former appears to be detectable only at the mRNA, but not protein level.^19,53^ Nevertheless, the tightness of our barrier model (mean TEER 112.3 Ω.cm^2^) was comparable to that reported at full maturity (i.e., 126 Ω.cm^2^ at 4 months^19^); the notion that barrier function may be developed well before 4 months is suggested by the literature evidence showing that TEER plateaus 2–3 weeks after plating on porous filters.^18,52^ Our TEER measurements were also in line with the reported 36–148 Ω.cm^2^ from isolated adult human RPE-choroid tissues.^21^

Future studies should test additional cell models, including human primary RPEs. They should also include *in vivo* experiments, for example, using translational animal models that simulate intraretinal effects of modified LDL, such as we have recently developed.^15^ Nevertheless, our current findings shed light on the effects of modified lipoproteins on RPEs and the action of fenofibrate, which are in concert with clinical observations.

Taken together, our data suggest that modified lipoproteins may contribute to outer BRB dysfunction, and that this effect can be mitigated by fenofibric acid. This finding may help explain the clinical benefits of fenofibrate in patients with DR. Elucidation of the pharmacologic mechanisms of fenofibrate in DR will help with the development of next-generation, better-targeted therapies for this disease.
